# Measuring the role of seagrasses in regulating sediment surface elevation

**DOI:** 10.1038/s41598-017-12354-y

**Published:** 2017-09-20

**Authors:** Maria Potouroglou, James C. Bull, Ken W. Krauss, Hilary A. Kennedy, Marco Fusi, Daniele Daffonchio, Mwita M. Mangora, Michael N. Githaiga, Karen Diele, Mark Huxham

**Affiliations:** 1 School of Applied Sciences, Edinburgh Napier University, Edinburgh, UK; 2 Department of Biosciences, Swansea University, Swansea, SA2 8PP UK; 3 U.S. Geological Survey, Wetland and Aquatic Research Center, 700 Cajundome Blvd., Lafayette, Louisiana 70506 USA; 4 School of Ocean Sciences, Bangor University, Anglesey, UK; 5 Biological and Environmental Sciences & Engineering Division, King Abdullah University of Science and Technology, Thuwal, Saudi Arabia; 6 Institute of Marine Sciences, University of Dar es Salaam, Zanzibar, Tanzania; 7 Kenya Marine and Fisheries Research Institute (KMFRI), Mombasa, Kenya; 8 St Abbs Marine Station, The Harbour, St Abbs, UK

## Abstract

Seagrass meadows provide numerous ecosystem services and their rapid global loss may reduce human welfare as well as ecological integrity. In common with the other ‘blue carbon’ habitats (mangroves and tidal marshes) seagrasses are thought to provide coastal defence and encourage sediment stabilisation and surface elevation. A sophisticated understanding of sediment elevation dynamics in mangroves and tidal marshes has been gained by monitoring a wide range of different sites, located in varying hydrogeomorphological conditions over long periods. In contrast, similar evidence for seagrasses is sparse; the present study is a contribution towards filling this gap. Surface elevation change pins were deployed in four locations, Scotland, Kenya, Tanzania and Saudi Arabia, in both seagrass and unvegetated control plots in the low intertidal and shallow subtidal zone. The presence of seagrass had a highly significant, positive impact on surface elevation at all sites. Combined data from the current work and the literature show an average difference of 31 mm per year in elevation rates between vegetated and unvegetated areas, which emphasizes the important contribution of seagrass in facilitating sediment surface elevation and reducing erosion. This paper presents the first multi-site study for sediment surface elevation in seagrasses in different settings and species.

## Introduction

Foundation species are organisms that structure their associated ecosystems, by moderating abiotic conditions and exerting strong influences on the whole biotic community^1^. Seagrasses are marine foundation species that form ecologically important habitats in coastal areas around the world^2,3^. They provide a range of ecosystem services, including habitat and nursery grounds for commercially important species, the regulation of water quality and the stabilisation of sediment^4–6^. Recently, the potentially large contribution of seagrass meadows (along with the other ‘blue carbon’ habitats of mangroves and tidal marshes) to global carbon sequestration and storage has also become apparent^7^. This long-term carbon storage relies on the ability of the plants to modify their environment. Coastal wetlands, including seagrass meadows, must elevate vertically such that the sediment surface keeps pace with rising sea level, in order to avoid falling below critical productivity and stability thresholds, which can lead to subsequent loss of stored carbon as the wetland deteriorates^8^.

Mangroves and tidal marshes can form effective natural coastal defences^9,10^. The coastal vegetation acts as a baffle for reducing wave and tidal energy, in addition to trapping sediment and raising the intertidal profile, thus directly contributing to coastal protection. Sediment stabilisation is often acknowledged as an important ecosystem function of seagrasses^3,11^, which in combination with other factors can lead to sustained elevation of the sediment surface in these habitats^12^. A well-developed network of rhizomes and roots anchors seagrasses into the sediment and directly contributes to buried organic carbon whilst the canopies reduce current speeds aiding the settlement of suspended allochthonous material^13–16^. These processes result in the accumulation of organically rich particles in a low oxygen environment, leading to carbon storage in the sediment, sometimes for millennia^17,18^.

Changes in sediment surface elevation are influenced not only by surficial processes of sediment deposition or erosion but also by subsurface processes, such as shallow subsidence and root expansion^19^; understanding elevation change over time requires accounting for both sets of processes. Sediment accretion rates and elevation change in seagrasses worldwide have been determined by various methods that consider historical and recent changes (Table 1). Mapping techniques (with e.g. Altus altimeter, Stanley compulevel, topographic surveys^20–22^) provide estimates of large scale and long-term changes in elevation and coverage, but lack the precision needed to track elevation changes annually. Radiometric dating methods using ^137^Cs, ^14^C, ^210^Pb and other isotopes to date marker depths^17,18,23–28^ can provide estimates of sedimentation rates over hundreds to thousands of years, whereas sediment traps^29^ measure sedimentation over days to a few months, but ignore root contributions to sediment binding. These traditional methods for measuring rates of sedimentation, however, provide neither direct estimates of surface elevation nor the opportunity to distinguish the contribution of subsurface processes to surface elevation change. Sediment accretion alone, whether measured over long or short time periods, is not equivalent to surface elevation change; assuming so can lead to overestimations of the vulnerability of some tidal wetlands^30,31^.

**Table 1:** Methods used to measure sediment elevation and accretion rates in seagrass meadows worldwide.

Method	Geographic area	Rate mm y^−1^ (±SE)	Habitat/Species present	Duration of study	Source
**Sediment Elevation**					
Rod Surface Elevation Tables (RSETs)	Oregon, USA (Valino Island)	10.08	Intertidal/*Zostera marina*, *Zostera japónica*	1 year	34
	Oregon, USA (Danger Point)	−5.28	Intertidal/*Zostera marina*, *Zostera japónica*	1 year	34
	Florida bay (Cross Bank, north side)	−7.7	*Thalassia testudinium, Halodule wrightii, Syringodium filiforme*	17 years	Frankovich, pers. comm.
	Florida bay (Cross Bank, south side)	13.5	*Thalassia testudinium, Halodule wrightii, Syringodium filiforme*	17 years	Frankovich, pers. comm.
Surface Elevation Tables (SETs)	Washington, USA (northern sites)	−5.1 (±1.27)	Intertidal/*Zostera marina*	4 years	35
	Washington, USA (southern sites)	−5.31 (±2.33)	Intertidal/*Zostera marina*	4 years	35
Altus altimeter	Bassin d’Arcachon, France	R = 8–32**	Intertidal/*Zostera noltii*	1 year	21
	Bassin d’Arcachon, France	−49**	Unvegetated area	1 year	21
Stanley Compulevel	Wadden sea	R = 5–7***	*Zostera marina* (planting unit)	3 months	20
	Wadden sea	<0.5	Unvegetated area	3 months	20
DGPS Trimble RTK topographic survey	Berre Point, France	from 10 to 30	*Zostera noltii*	14 months	22
	Berre Point, France	from −30 to −10	Unvegetated areas	14 months	22
NK	Rhode Island, USA	12.5*	*Zostera marina*	2 years	63
NK	Rhode Island, USA	−7.5*	Unvegetated plots	2 years	63
**Sediment accretion**					
^14^C	Seto Inlad Sea, East Asia	0.9 (±0.28)	*Zostera marina*	NA	27
	Seto Inlad Sea, East Asia	0.32	Unvegetated area	NA	27
	Ishigaki Island, Southeast Asia	1.23	*Enhalus acoroides*	NA	27
	Southern Thailand	0.82	*Enhalus acoroides, Thalassia hemprichii*	NA	27
	Ischia, Italy	1.65	*Posidonia oceanica*	NA	17
	Culip, Spain	0.61	*Posidonia oceanica*	NA	17
	Port-Lligat, Spain	4.14	*Posidonia oceanica*	NA	17
	Campello, Spain	2.03	*Posidonia oceanica*	NA	17
	Tabarca 1, Spain	1.14	*Posidonia oceanica*	NA	17
	Tabarca 2, Spain	1.88	*Posidonia oceanica*	NA	17
	Medes, Spain	0.79	*Posidonia oceanica*	NA	17
	Port Lligat, Spain	1.3	*Posidonia oceanica*	NA	25
	Port Lligat, Spain	1.1	*Posidonia oceanica*	NA	24
	Talamanca Cove, Spain	2.3	*Posidonia oceanica*	NA	26
	Pujols Cove, Spain	1.7	*Posidonia oceanica*	NA	26
	Mellieha Bay, Malta	4.9	*Posidonia oceanica*	NA	28
	Salina Bay, Malta	4	*Posidonia oceanica*	NA	28
	Sydney, Botany Bay	R = 4.7–9.9	*Posidonia australis, Zostera capricornii*	NA	18
	Oyster Harbor, Australia	0.49	*Posidonia australis*	NA	28
	Waychinicup Inlet, Australia	0.43	*Posidonia australis*	NA	28
	Big Lagoon, Australia	0.51	*Posidonia australis*	NA	28
	Port Pirie, Australia	0.13	*Posidonia australis*	NA	28
	Port Broughton, Australia	2.5	*Posidonia australis*	NA	28
	Cockburn Sound, Western Australia	R = 0.6–1.3	*Posidonia sinuosa*	NA	26
^210^Pb	Florida Bay	9 (±7)	*Thalassia testudinum*	NA	23
Sediment traps	Western Baltic	2.2	*Zostera marina*	8 months	64
	Fanals Point, Spain	2	*Posidonia oceanica*	14 months	29
	Fanals Point, Spain	3	Unvegetated area	14 months	29
Foraminifera	Spencer Gulf, Australia	R = 0.15–0.25	*Posidonia australis*	NA	65

^*^Ganthy *et al*. reported that between February and September of 2009, sediments were accreted at all seagrass stations (+41 mm, +16 mm, +15 mm for high density HD, medium density MD and low density LD plots respectively) whereas unvegetated showed minimal change ( + 3 mm). Between September 2009 and February 2010, sediments eroded at all seagrass stations (−9 mm, −6 mm, −7 mm for HD, MD and LD respectively), whereas the unvegetated station showed a strong erosion of −54 mm.

**Harlin *et al*. reported 2.5 cm of accretion in seagrass plots, and 1.5 cm of erosion in denuded plots in the course of 2 years.

***Measurements reported for the growing season.

NK: Not known, NA: Not Applicable, R: Range.

Patterns of elevation and accretion have been extensively studied in mangroves and tidal marshes around the world using Surface Elevation Tables (SETs) and closely related Rod Surface Elevation Tables (RSETs)^32^. This approach measures above and below ground processes and their effects on surface elevation. It allows very precise measurements - to within 1.5 mm^33^ - that can be regularly taken – giving data that may inform ecosystem responses to short-term events, including storms, disease and grazing, but which may also be continued over years or decades. Hence, the RSET method complements and extends other approaches, and has been recommended as a global standard for measuring wetland vulnerability to climate change given the right spatial coverage and timeframes of monitoring^32^. Despite the similarities between mangroves, tidal marshes and seagrass meadows, and the advantages of the RSET methodology, very few studies have so far used RSETs or similar methods in seagrasses^34,35^. Testing the applicability of approaches that provide similar insight to seagrasses as those gained from RSET approaches in other wetlands, in a range of settings, was a key objective of the current work. In addition, most studies on sedimentation and elevation in seagrass meadows have not used appropriate controls, making it difficult to understand the relative impacts of seagrass and general geo-morphological settings. Hence comparing rates with control sites was another important goal.

Seagrass ecosystems face numerous anthropogenic threats including increasing storms, seawater warming, sea level rise, eutrophication and mechanical damage from boating and fishing. At an estimated average decline of 1.5% of global distribution per year since the time of the earliest records in the late 1800s^36^, they are suffering the fastest rate of loss of the three blue carbon systems, and 65% of seagrass systems worldwide are thought to be degraded^37^. Such estimates are however uncertain given the relative paucity of studies on seagrass distribution and condition, compared with mangroves and tidal marshes, and the accelerating rates of decline (median rate of decline <1% per year before 1940, 5% per year after 1980). Degradation and loss of seagrasses lead to diminution and loss of their ecosystem services. This loss of ecosystem services value may be rapid (for example with fisheries habitat provision) or delayed (for example carbon may remain stored below-ground for some time after degradation begins under some circumstances^38^). The temporal relationship between seagrass degradation and loss, and coastal protection is currently unknown.

There is, therefore, a need to test the role of seagrass meadows on surface elevation change and sediment retention, and to explore the utility of a standard method, using sediment pins, for measuring elevation change in seagrass settings. Building capacity to measure sedimentary processes and surface change in seagrass meadows will increase our understanding of these key processes, and may allow us to model seagrass vulnerability and management needs in the face of sea level rise as has been achieved for mangroves and tidal marshes^31^. The present study aims to assess to what extent seagrass meadows contribute to sediment deposition and stability, determining their role on sediment dynamics, in a range of settings and locations. More specifically, the objectives were to: i) test the utility of sediment pins approach in determining the rate of surface elevation change in seagrass and unvegetated plots of different species and in different locations, ii) examine seasonal and geographical patterns in surface elevation change, and iii) compare these to published sedimentation rates.

## Results

### Cumulative sediment surface elevation and rates

#### Scotland

**Figure 1:**
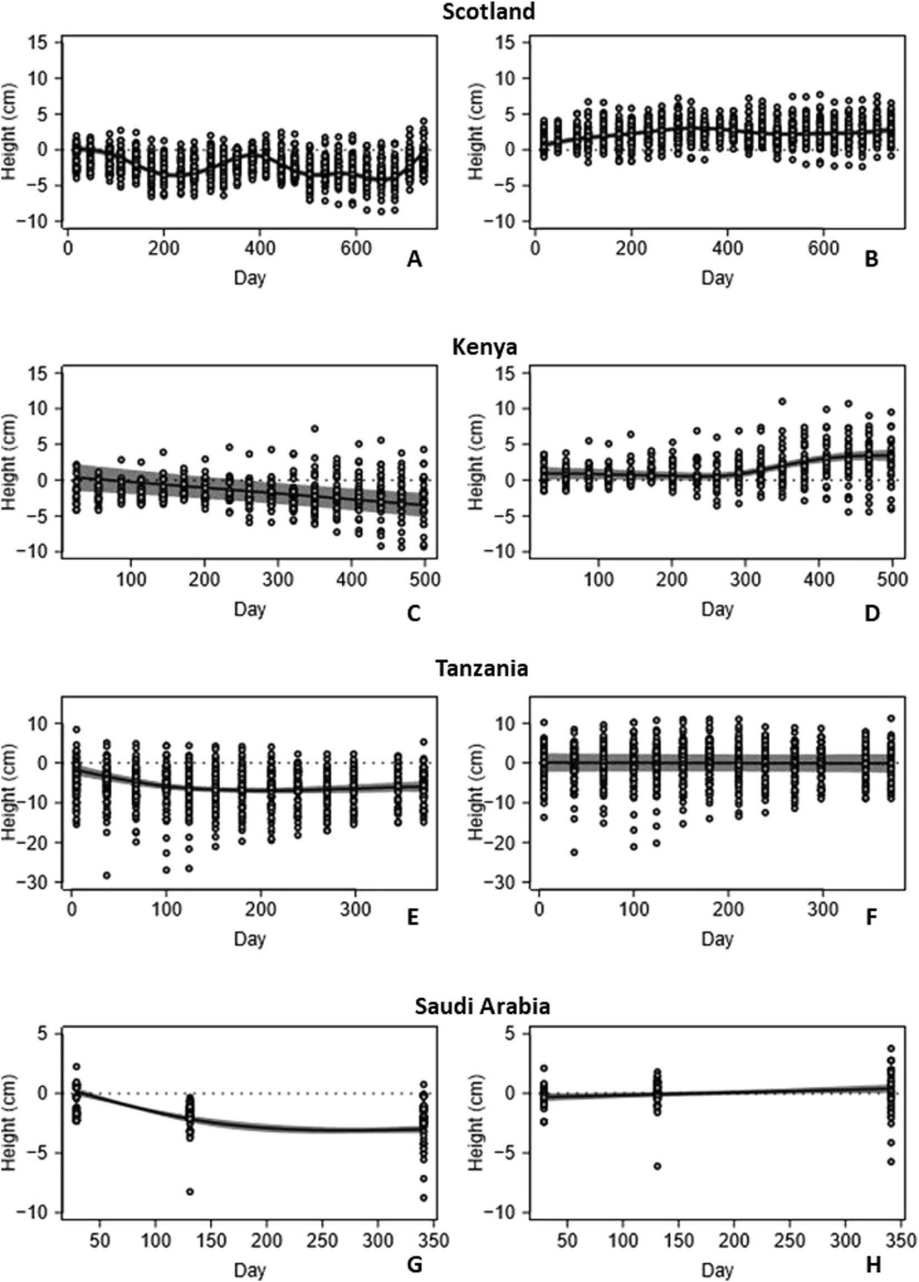
Cumulative surface elevation change at unvegetated (left column; **A**,**C**,**E**,**G**) and seagrass plots (right column; **B**,**D**,**F**,**H**) in Scotland, Kenya, Tanzania and Saudi Arabia (from the top to the bottom). Solid lines show mean trajectories, with shaded area representing 95% confidence intervals. A horizontal dotted line indicates zero net height change.

At plots where seagrass was absent, there was an apparently cyclical trend in sediment surface elevation through the course of the study (Fig. 1A). This resulted in no net annual change in the average height of sand across the length of the study, but with pronounced losses in winter, and accumulation in summer. In this case, a sinusoidal function with a period of one year was a significantly better descriptor of the data than a linear function (Likelihood Ratio (L.R.) = 199, df = 1, p < 0.001). Conversely, at plots where seagrass was present (Fig. 1B), there was a statistically significant increase in sediment elevation across the whole study period (mean = 9.01 (SE = 2.17) mm per year, t = 4.16, df = 1, p < 0.001). Overall, 99.4% of variance was explained by the fixed effects of time and treatment. Of the remainder, a small but statistically significant amount of variation was explained by random differences between rods, within plots (variance component = 0.03%, L.R. = 12.6, df = 1, p < 0.001), and by random differences between plots (variance component = 0.04%, L.R. = 12.7, df = 1, p < 0.001), with 0.53% of variance unexplained. Sediment height measured with 2 m rods was not significantly different from the one measured with standard pins (F = 2.33, df = 1, p = 0.128), indicating that the rods do not change vertical position in the sediment.

#### Kenya

At plots where seagrass was absent (Fig. 1C), there was a significant linear reduction in sand height throughout the study (mean = 34.7 (SE = 7.26) mm per year, t = 5.19, df = 1, p < 0.001). Where seagrass was present (Fig. 1D), there was statistically significant non-linear trend (L.R. = 24.6, df = 7, p = 0.0089) resulting in an average sand height gain of 33.7 (SE = 7.08) mm over the length of the study. There was no evidence of an annual cyclical pattern in this non-linear trend (L.R. = 0.719, df = 1, p = 0.40). At this site, 61.8% of variance was explained by time and treatment. Of the remainder, a small but statistically significant amount of variation was explained by random differences between rods, within plots (variance component = 1.26%, L.R. = 56.5, df = 1, p < 0.001), and by random differences between plots (variance component = 12.0%, L.R. = 121, df = 1, p < 0.001), with 24.9% of variance unexplained.

#### Tanzania

At plots where seagrass was absent, there was a statistically significant non-linear trend (L.R. = 22.0, df = 7, p = 0.0026) resulting in an average sediment elevation reduction of 44.7 (SE = 12.6) mm over the length of the study (Fig. 1E). There was no evidence of an annual cyclical pattern in this non-linear trend (L.R. = 3.67, df = 1, p = 0.055). Where seagrass was present (Fig. 1F), there was no significant trend in sediment elevation change across the study period (t = 0.821, df = 1, p = 0.41). Here, 25.2% of variance was explained by the fixed effects, with 0.01% of variance attributed to random differences between rods (L.R. = 31.3, df = 1, p < 0.001), 24.6% as variance between plots (L.R. = 208, df = 1, p < 0.001), and 50.1% of variance unexplained.

#### Saudi Arabia

At plots where seagrass was absent, there was a statistically significant non-linear trend (L.R. = 518, df = 7, p < 0.001) resulting in an average sediment elevation reduction of 30.9 (SE = 4.52) mm over the length of the study (Fig. 1G). There was insufficient temporal resolution to test for seasonal trends at this site. At plots where seagrass was present (Fig. 1H), there was a statistically significant linear increase in sediment elevation (mean = 7.84 (SE = 1.48) mm per year, t = 5.31, df = 1, p < 0.001). Finally, at this site, 97.0% of variation was explained by the effects of time and treatment. Here, random variation between rods and plots was not statistically significant (variance components <0.01%) and the remaining 3% of variance attributed to residual error.

### Comparison with published surface elevation rates

**Figure 2:**
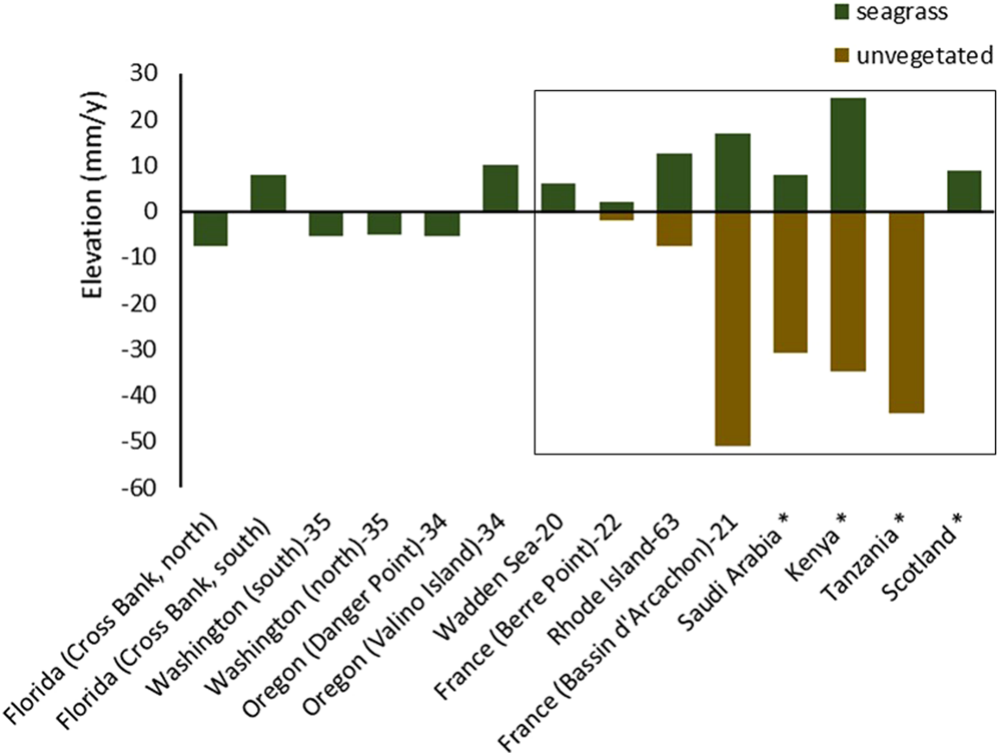
Sediment elevation rates (mm/y) in seagrass and unvegetated plots compiled from the literature (marked with the reference number) and this study (marked with an asterisk). The studies that reported sediment elevation rates for both seagrass and unvegetated plots are enclosed in the square. Note that the control plots in the Wadden Sea showed an erosion of <0.5 mm/y, whereas in Tanzania and Scotland, there was no net annual sediment elevation change for seagrass and control plots respectively.

The literature review found ten other studies that produced sediment elevation rates data; although only four of these produced directly comparable data allowing comparisons with unvegetated controls. Previous studies in seagrasses on sediment elevation, using multiple methods (SET, RSET, Altus altimeter, DGPS Trimble RTK and Stanley compulevel) and sampling a wide range of elevations (subtidal to high intertidal) have yielded variable results (Fig. 2; Supplementary Information) (for the whole dataset see Table 1). When pooling all available data from the literature and the present study, seagrasses are facilitating sediment surface elevation at a rate of 5.3 (SE = 2.69) mm per year, whereas in unvegetated plots (where available), sediment is eroding at a rate of 21.3 (SE = 7.33) mm per year. The overall effect of seagrasses by averaging the within-site mean differences, where both seagrass and unvegetated plots were reported, was 31.2 (SE = 9.57) mm per year (Supplementary Information).

### Water motion

**Figure 3:**
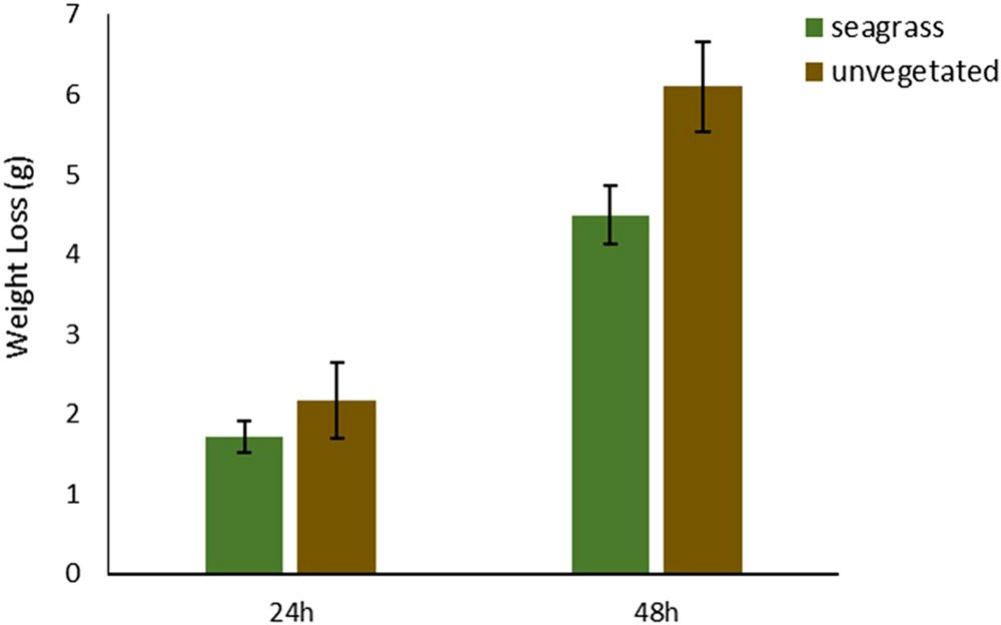
Weight loss of clod cards in seagrass and unvegetated plots after 24 h and 48 h (Error bars: 95% confidence intervals).

The weight loss of the plaster blocks deployed in unvegetated plots was significantly higher than seagrass plots after 48 h (df = 1, t = −4.67, p < 0.01), indicating that hydrodynamic energy was lower at seagrass patches compared to unvegetated areas. This was not observed, however, 24 h after deployment; in that case the weight loss of the plaster blocks of control plots was not significantly different from seagrass plots (df = 1, t = −1.69, p = 0.152). More specifically, the weight of the plaster blocks in seagrass plots was reduced by 14% and 32% after 24 h and 48 h respectively, whereas in unvegetated areas, that reduced by 17% and 42% (Fig. 3).

## Discussion

The present study demonstrates that seagrass meadows can stabilise sediments and help to facilitate surface elevation. This occurs at sites in very different settings (both inter and subtidal, and tropical and temperate) and with a wide range of seagrass species. Importantly our results also allow comparisons with unvegetated control areas (chosen for their proximity and similarity to the seagrass plots) which reveal how seagrasses can drive surface elevation even in settings where erosion is occurring without them. The impact of seagrasses is remarkably strong, with an average difference of 31 mm per year in elevation rates between seagrass and unvegetated areas; in comparison, surface elevation change in natural mangroves ranges between −5.8 to 6.3 mm per year^8,39^. This much greater impact may be related to the much greater hydrodynamic energy and longer (or permanent) periods of submersion in seagrasses compared with mangroves.

Sediment trapping and stabilisation will cause much of this seagrass-mediated elevation. The Plaster of Paris clods showed significant reductions in current speeds at the Scottish site, and work at the Kenyan site gave similar results (Githaiga, pers. comm.), which accords with previous studies showing that seagrasses reduce current and wave energy^40,41^. Sediment accumulation in seagrass beds results from a balance between deposition of suspended sediment and resuspension. The capacity of seagrasses for balancing these two processes is highly dependent on the development stage and health of the plants, as well as local hydrological conditions. Sediment stabilisation by seagrasses has been generally attributed to seagrass canopy properties^15,16,42^, which decrease the physical stress on the sediment-water interface, thereby creating and maintaining a stable hydrodynamic state^43^. Intriguingly, however, the Scottish site shows sediment stabilisation and retention even during winter, when above-ground biomass is sparse and when the control plots show strong seasonal patterns of erosion. This suggests that canopy biomass and form alone are not sufficient to explain sediment retention and that surface processes (possibly involving associated flora) and subsurface effects of roots and rhizomes are also important^44,45^. Previous studies have demonstrated that seagrasses with low canopies highly variable in biomass and cover, or strongly reduced canopies caused by grazing by turtles, can still prevent the sediment from eroding^46^. Data from Kenya (Githaiga *et al*., unpublished) show enhanced bioturbation following seagrass removal; so faunal impacts may also play a part.

One of the strengths of the combined RSET and marker horizons (MH) approach is that it allows for discrimination between sediment accretion and subsurface processes in their contributions to surface elevation or subsidence^47^. Unfortunately our attempts to measure both of these processes simultaneously were largely unsuccessful; using feldspar and other materials for MHs proved difficult or impossible in these permanently waterlogged or submerged plots, where even if an MH could be successfully established it was often washed rapidly away. We were able to measure elevation rates in four very different sites, using cheap materials that could be simply installed and replicated. The structural support for a standard RSET platform can create scouring (e.g. at the Collver Point study site^34^), leading to holes of a few centimetres deep to cones of depression (Frankovich, pers. comm). Hence this approach is unsuitable for seagrass habitats, whilst our SECP methodology did not cause obvious scouring. However, we are not able to distinguish between surface and subsurface processes with our approach because we could not combine it with an acceptable MH method, and we suggest that future work should combine other types of sediment trapping with rods in order to do this.

The rates of elevation recorded here lie within the range of values reported from other studies (Table 1; Supplementary Information), although the addition of data from unvegetated sites emphasizes the large relative average contribution of seagrass, suggesting the effects of seagrass are much greater than implied by elevation rates in seagrass plots alone. Previous meta-analysis from Duarte *et al*.^48^ using three estimates available at the time, indicates that subsidence occurs at seagrass meadows with sediment eroding at a rate of 0.08 mm per year; in comparison our meta-analysis suggests that seagrasses facilitate sediment deposition at a rate of 5.3 mm per year. Also, the elevation rates reported here are generally higher than values for accretion. Whilst Orem *et al*.^23^, using ^210^Pb, revealed accretion rates of 9 ± 7 mm per year (mean ± SE), the sediment trapping by Gacia & Duarte^29^ showed accretion of 2 mm per year, and studies using^14^C dating suggest much lower accretion rates, of 1.6 ± 0.3 mm per year (mean ± SE), at the millennial time scale. Such a discrepancy (with long term accretion rates lower than elevation estimates) is common in the coastal wetlands literature, and may reflect the impacts of long-term geological compaction^32^. The mean difference between vegetated and unvegetated areas that we recorded here – 31 mm per year – is clearly unlikely to be representative over long time periods, since it would imply total erosion of unvegetated sites. Rather it captures some of the short term changes in these dynamic systems and gives a strong impression of the powerful short term effects of seagrass in damping sediment movement. Using radiosotopes for estimates over either centennial (^210^Pb) or millennial (^14^C) timescales gives data relevant to long term retrospective or predictive understanding but does not accurately capture current conditions and short-term ecological and biogeophysical drivers. Furthermore, the sediment mixing and erosion that is common in seagrass sites, and which itself is influenced by seagrass characteristics, can obscure or potentially confound the results from isotope work. Also, there has been no study to date of spatial variability in accumulation rates in seagrass meadows and often dating of sediments where an accumulation rate cannot be provided is omitted from published datasets. In common with RSETs in other coastal wetlands, our modified approach here gives an estimate of integrated elevation change over years to (potentially) decades, but would need to be combined with other approaches to expand the timeframe beyond that.

Seagrass meadows have suffered widespread declines and degradation over the past century, and these trends are predicted to continue and accelerate^36,49,50^. Given the powerful impact on surface elevation through sediment accretion and retention that is demonstrated here, predicted seagrass loss will have major impacts on the stability of coastal sediments and on coastal geomorphology. The average organic carbon stock in seagrass meadows has been estimated to be 139.7 Mg C ha^−1^ within the top metre of the sediment making them important global carbon sinks^48,51^. Since 95% of C in seagrass is stored below ground in sediments, the loss of sediment stabilisation and elevation functions would remove future sequestration potential as well as threaten the release of carbon already buried in these carbon dense ecosystems.

While global mean sea level has been gradually increasing for at least 18,000 years, this trend has accelerated in the last 20 years in response to climate change^52^. In an era of rising seas, a substantial shift in suitable habitat for seagrasses will occur. At intertidal or shallow subtidal areas, seagrasses will migrate shoreward^53,54^, if excessive coastal development or seawalls do not already prevent this. In deeper areas, the reduced light availability will cause substantial seagrass losses, which will be intensified if there are not adjacent suitable areas for colonisation. Identifying areas where seagrasses could regulate surface elevation and providing managers with appropriate tools to monitor the degree of resilience or vulnerability, would be key elements for seagrass conservation as well as restoration projects. The present study represents an important first step in assessing the role of seagrasses in controlling sediment surface elevation and calls for the development of a global monitoring network for seagrasses similar to that for mangroves and saltmarshes. This will co-ordinate and facilitate systematic and long-term measurements across a broad range of geographical settings for better understanding of the future status of seagrass meadows and their continued provision of biodiversity and ecosystem functions in the wake of sea level rise.

## Materials and Methods

### Study Sites

**Figure 4:**
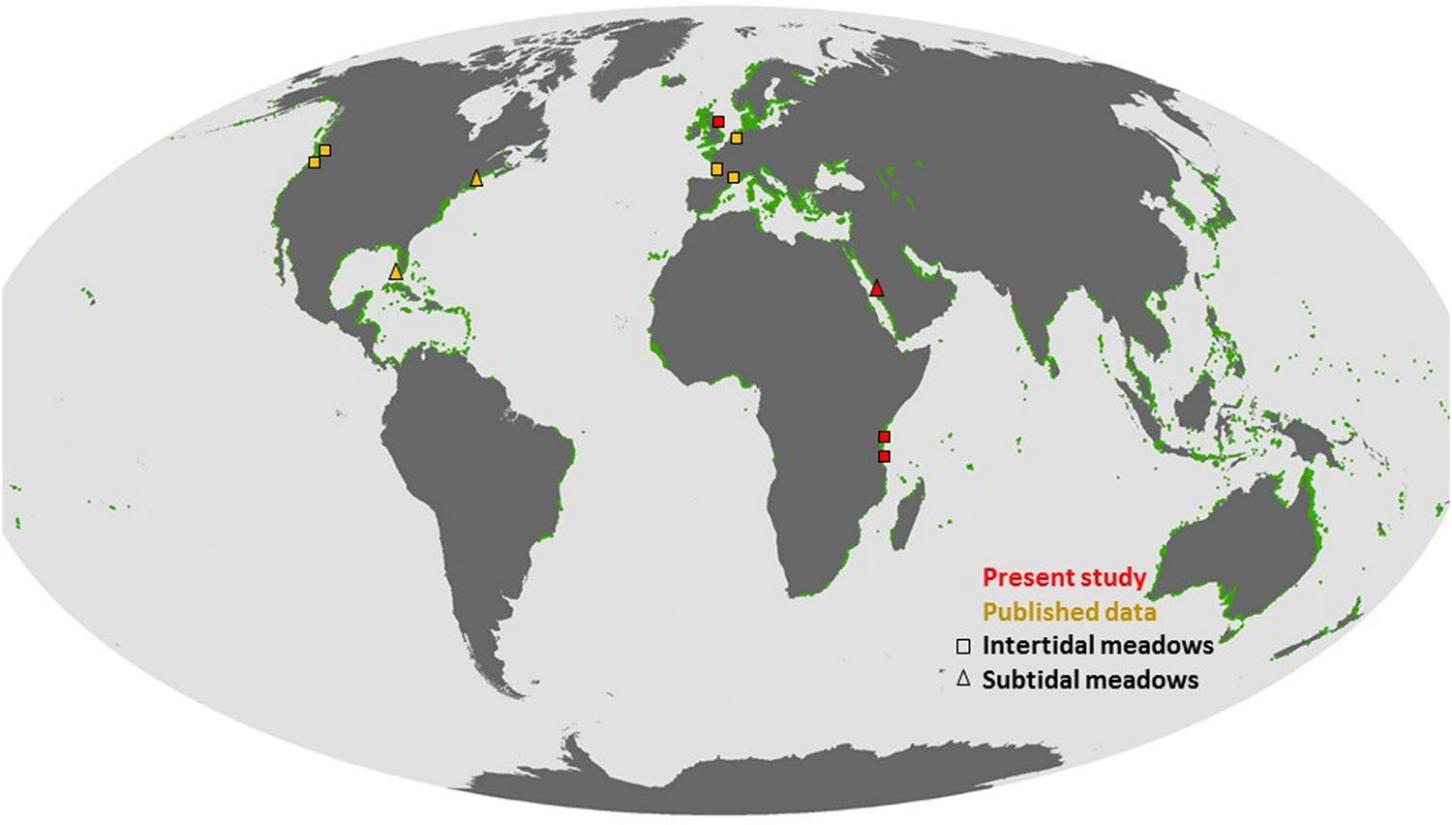
Map of the locations where sediment elevation rates have been reported, from our sites and published studies, both for intertidal and subtidal seagrass meadows. The background map shows the latest dataset for the global seagrass distribution (available from UNEP-WCMC, Short FT (2016). Global distribution of seagrasses (version 4.0). Fourth update to the data layer used in Green and Short (2003). Cambridge (UK): UNEP World Conservation Monitoring Centre^62^. http://data.unep-wcmc.org/datasets/7).

The study was conducted in four locations: Scotland, Kenya and Tanzania (intertidal sites), and Saudi Arabia (subtidal site) (Fig. 4). In Scotland, the site is located in Drum Sands (55°59′N 3°19′W), in the macrotidal Firth of Forth, with only intertidal *Zostera noltii* present. Seagrass patches differ in size and shoot density (ranging from 105 to 1881 shoots m^−2^), with an average shoot length of 179 ± 9 mm (±SE). The local microtopographic relief retains water during low tides, with a mosaic of mounds with seagrass growing on the top and generally unvegetated pools. In Kenya, the site is located in mesotidal Gazi Bay (4°25′S 39°30′E), on the southern coast, in Kwale County. There are twelve seagrass species present at the bay (*Cymodocea rotundata, Cymodocea serrulata, Enhalus acoroides, Halodule uninervis, Haludule wrightii, Halophila minor, Halophila ovalis, Halophila stipulacea, Syringodium isoetifolium, Thalassia hemprichii, Thalassodendron ciliatum, Zostera carpensis*) occurring either as monospecific or multispecific stands. The study area, which was located between the two creeks of the bay, is dominated by *Thalassia hemprichii* and *Enhalus acoroides* with unvegetated patches found within the meadow. In Tanzania, the site is located in the mesotidal Unguja Ukuu (6°19′S 39°22′E) on the southwestern side of Unguja Island of Zanzibar, with five seagrass species present (*Cymodocea rotundata, Halodule uninervis, Halophila ovalis, Thalassia hemprichii, Thalassodendron ciliatum*) and patches of unvegetated pools, mud and sand flats. Seagrass plots contained a mixture of species and control plots were placed in the bare patches within the mixed meadows. Whilst the seagrasses occur on top of the small sediment mounds in the Firth of Forth, the seagrasses in Gazi Bay and Unguja Ukuu generally occur in shallow pools between unvegetated mounds. In Saudi Arabia, the site is located in the mesotidal lagoon of Al Qadeimah, near the King Abdullah Economic City, north of Jeddah (22°23′N 39°7′E). The dominant species here is *Enhalus acoroides* (shoot density: 38 ± 4.8; mean ± SE) with minor occurrence of *Halodule uninervis* and *Cymodocea serrulata*. Unvegetated control plots were located 1 km away (southward) at 22°22′N 39° 7′E.

### Sediment Surface Elevation measurements- Establishment of Surface Elevation Change Pins (SECPs)

**Figure 5:**
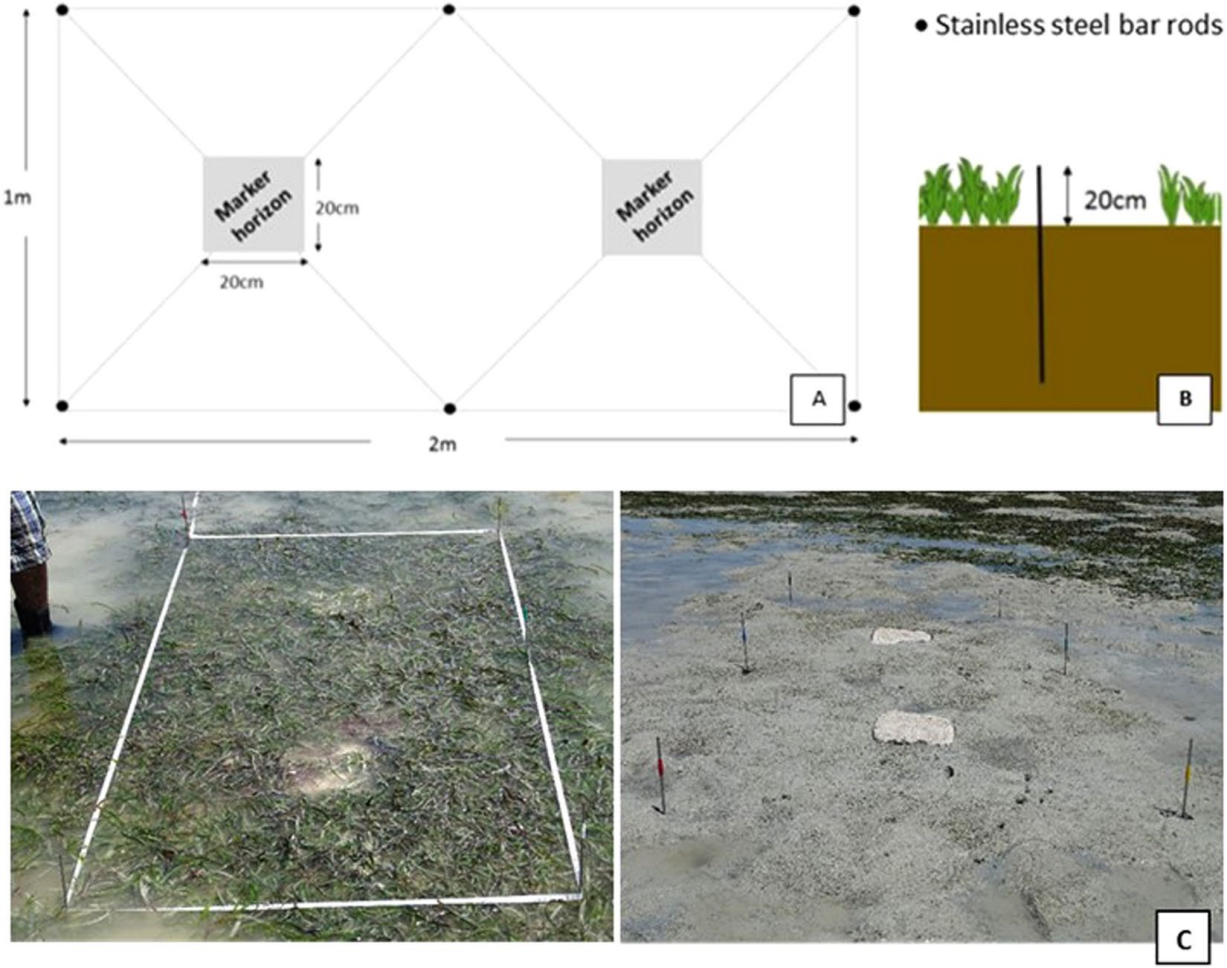
(**A**) Top view of the SECP plot layout, (**B**) Vertical position of rods, (**C**) Seagrass and unvegetated (control) plots set-up in Tanzania.

Ten 1 × 2 m plots, five treatment plots (with seagrass) and five control plots (no seagrass), were established in May and June of 2014 in Scotland and Kenya respectively, and March and May of 2015 in Saudi Arabia and Tanzania respectively. Each plot consisted of six stainless steel bar rods (5 mm diameter, 1.2 m length) protruding ~20 cm above the sediment. At the Scottish site, we also deployed longer rods of 2 m length adjacent to the standard rods (1.2 m) at a control (no seagrass) plot in order to test whether subsidence of rods would occur. The layout of a sampling plot is presented in Fig. 5. Elevation measurements were collected monthly in Scotland, Kenya and Tanzania and seasonally in Saudi Arabia, placing a light plastic washer (ID: 1.5 cm, OD: 4 cm) over the rod so that it rested gently on the sediment and measuring upwards from this to the tip of the exposed rod as an integrated measure of sediment surface.

Marker horizons (MHs) were also deployed, two on each plot, to quantify sediment surface deposition. Different materials were used depending on the specific settings of each site. For the intertidal sites (Scotland, Kenya and Tanzania), feldspar clay was used; a material that forms a cohesive layer once it gets wet and is easily distinguishable from the surrounding sediment. A polysterene trashcan with the bottom cut off was used to define the borders of the marker horizon plot (20 cm × 20 cm) and the feldspar clay was laid on the sediment surface to a thickness of 1 cm. In Kenya, dyed sand was also used in addition to feldspar clay. For the subtidal site (Saudi Arabia), plastic louvers (or eggcrates) of the same dimensions (20 cm × 20 cm) were used as an alternative to feldspar clay and dyed sand, anchored in the sediment with long pegs. Despite the use of different materials, MHs proved to be difficult to sample, as they were washed rapidly away in the case of feldspar clay/dyed sand or displaced by burrowing animals in the case of plastic louvers.

Surface Elevation Change Pins serve here as a modified design of the previously developed, Shallow RSETs^33^, which if used on our four study sites would be cost-prohibitive, highly visible (and thus at risk of interference) and bulky, and therefore potentially influencing the processes we aimed to measure. More specifically, Shallow RSETs make use of a firm base with four legs installed to more shallow depths than standard RSETs in order to represent surface elevation change relative to the root zone and shallow geomorphological processes dominant in seagrasses^35^. Originally, the Shallow RSET was configured by Cahoon *et al*.^33^ for insertion to 35 cm to canvass the root zone of focal emergent wetlands. In our design, we canvass a full metre of sediment to relate to standard Blue Carbon assessment depths in seagrasses^51^ and to prevent eddy influences that might result from Shallow RSET platform-associated erosion during peak ebb and flow tidal events in seagrasses. Pin networks have been used experimentally as cost-effective means to measure wetland soil surface elevation change in remote locations in Micronesia, Sri Lanka and Kenya^55–57^ and have been especially critical in disentangling the influences of root zone versus surface depositional processes in those focal wetlands.

### Water motion

To evaluate the wave reducing effect of seagrass patches, we compared weight loss of Plaster of Paris blocks, ‘clod cards’, deployed at the ten plots (seagrass and unvegetated) described above at the Scottish site. Relative weight loss by dissolution of the plaster is a proxy for hydrodynamic forcing and integrates effects from tidal currents and waves^58–60^. The blocks were moulded using Plaster of Paris and made in ice cube trays. They were attached to plastic rings with silicon, which was applied to the base of each block, and then the block-ring complex was fastened with cable ties onto wooden sticks. The sticks were inserted in the sediment (with plaster of paris blocks just above the sediment surface) and left *in situ* for 24 hours (2 tides) and 48 hours (4 tides) in August 2015. The blocks were weighed before and after each deployment, after drying at 40 degrees Celsius until constant weight.

### Sediment elevation and accretion rates of seagrasses globally

We compiled published data available on sediment elevation and accretion rates in seagrass meadows and adjacent unvegetated sediments where available. The data were collated from the literature by conducting a Boolean search in Web of Knowledge and Google Scholar using the word combinations “seagrass”, “elevation rate” and “accretion rate”. From each study, the method used, geographic area, habitat type and species present, length of study and the source are reported.

### Statistical Analysis

We quantified changes in sediment height through time using Generalised Linear Models (GLMs) and Generalised Additive Models (GAMs). Height (cm) of exposed rod was fitted as the response variable, with ‘treatment’ (presence or absence of seagrass) included as a categorical explanatory variable. Time was modelled as a continuous explanatory variable using smoothing splines (GAMs) at all study sites. Where non-linear trends through time were identified, we also fitted statistically linear sinusoidal functions (GLMs) to assess seasonal effects. In all cases, variation due to the replication structure of the experimental design was fitted as nested random effects in a mixed-effects modelling framework, with replicate rods nested within replicate plots. Finally, we modelled temporal autocorrelation between monthly sampling points as a first order autoregressive process (AR1). Gaussian error distribution was confirmed by visual inspection of residual Q-Q plots and we tested for heteroscedasticity of residuals using Breusch-Pagan tests. Where either assumption was not met, we refitted models on natural logarithm-transformed absolute height values (since logarithms of negative numbers are invalid) and assumptions were met.

All statistical analysis was performed using R v3.3.2, with additional functions within the ‘mgcv’ package^61^.

### Data availability

The datasets generated and analysed during the current study are available from the corresponding author^63–65^.

## Supplementary information

Supplementary information
