# Supplementary material for: Measuring the role of seagrasses in regulating sediment surface elevation
Source: Sci Rep. 2017 Sep 20;7:11917. doi: 10.1038/s41598-017-12354-y (PMC5605501; doi:10.1038/s41598-017-12354-y)
Supplement: Supplementary file 1 — Supplementary information [file 41598_2017_12354_MOESM1_ESM.pdf]

SUPPLEMENTARY INFORMATION

Measuring the role of seagrasses in regulating sediment surface elevation.

Maria Potouroglou[1*], James C. Bull[2], Ken W. Krauss[3], Hilary A. Kennedy[4], Marco Fusi[5], Daniele Daffonchio[5], Mwita M. Mangora[6], Michael N. Githaiga[1,7], Karen Diele[1,8], Mark Huxham[1]

[1] School of Applied Sciences, Edinburgh Napier University, Edinburgh, UK. [2] Department of Biosciences, Swansea University, Swansea, SA2 8PP, UK. [3] U.S. Geological Survey, Wetland and Aquatic Research Center, 700 Cajundome Blvd., Lafayette, Louisiana, 70506, USA. [4] School of Ocean Sciences, Bangor University, Anglesey, UK. [5] Biological and Environmental Sciences & Engineering Division, King Abdullah University of Science and Technology, Thuwal, Saudi Arabia. [6] Institute of Marine Sciences, University of Dar es Salaam, Zanzibar, Tanzania. [7] Kenya Marine and Fisheries Research Institute (KMFRI), Mombasa, Kenya. [8] St Abbs Marine Station, The Harbour, St Abbs, UK. *e-mail: m.potouroglou@napier.ac.uk

Sediment surface elevation rates at the study sites, and those compiled from the literature and this study.

Sediment elevation rates in mm per year (apart from the values in bold) at the study sites. The seagrass plots in Kenya and the control plots in Tanzania, Saudi Arabia showed a non-linear trend over the length of the study, and thus rates are originally reported in the text as such. However, they are given here under the table in mm per year for direct comparisons in future studies.

	Sediment elevation rates	
	Seagrass<br>Mean (SE)	Unvegetated<br>Mean (SE)
Scotland	9.01 (2.17)	0
Kenya	**33.7 (7.08)**[1]	34.7 (7.26)
Tanzania	0	**44.7 (12.6)**[2]
Saudi Arabia	7.84 (1.48)	**30.9 (4.52)**[3]

[1] 24.6 (SE = 5.29) mm per year

[2] 43.9 (SE= 12.4) mm per year

[3] 33.1 (SE= 4.84) mm per year

Sediment elevation rates compiled from the published literature and the results of this study.

	Sediment elevation rates (mm/y)		
	Mean	SE	N
Seagrass meadows where unvegetated plots were reported	9.9	2.85	8
Unvegetated plots	-21.3	7.33	8
All seagrass sites	5.3	2.69	14
Difference derived from seagrass and unvegetated plots, when both are reported at the same study	31.2	9.57	8
